# Impact of COVID-19 infection on laboratory and clinical outcomes of ovarian stimulation using antagonist protocol

**DOI:** 10.3389/fmed.2025.1674189

**Published:** 2025-12-19

**Authors:** Yu Yan, Mengxi Guo, Xiaojun Chen, Qing Zhang, Li Wang, Meiling Zhang, Min Wang, Wen Li, Yu Tao

**Affiliations:** 1 Center for Reproductive Medicine and Fertility Preservation Program, International Peace Maternity and Child Health Hospital, School of Medicine, Shanghai Jiao Tong University, Shanghai, China; 2 Shanghai Key Laboratory of Embryo Original Diseases, Shanghai, China

**Keywords:** COVID-19, IVF outcome, embryonic development, ovarian response, controlled ovarian stimulation, fertility evaluation

## Abstract

**Introduction:**

The COVID-19 profoundly impacted human reproduction, and provoked concerns regarding its potential influence on the assisted reproduction treatment outcomes. The current study designed to explore the impact of COVID-19 infection on the laboratory and clinical outcomes of patients who underwent controlled ovarian stimulation (COS) with antagonist protocol.

**Methods:**

This strictly self-controlled study included 134 patients who underwent repeated oocyte retrieval at the reproductive medicine center of International Peace Maternity and Child Health Hospital of China Welfare Institute between January 1, 2022 and December 31, 2023. Sixty six patients were contracted COVID-19 between their first and second COS cycles, and 68 patients were uninfected controls. We evaluated the laboratory outcomes, including oocyte yield and the rate of MII oocyte, fertilization, usable embryo, and high-quality embryo, through both inter-group (infected vs. non-infected) and intra-individual (before vs. after infection) comparisons.

**Results:**

The baseline characteristics were comparable between infection and non-infection groups. Ovarian reserve and response profiles demonstrated no statistically significant differences between the first and second COS cycles within either group. In the non-infection group, both the available and high-quality embryo rate showed significant improvement in the second COS cycle. In the infection group, although the blastocyst formation rate was significantly higher after COVID-19 infection (*p* = 0.011), the high-quality embryo rate did not differ significantly between the pre- and post-infection cycles. We also stratified the infection group into five subgroups based on the interval from infection to oocyte pick-up. An increase in both total and high-quality blastocyst rates was observed in the subgroup with an interval of 181–240 days post-infection. The pregnancy outcomes were similar between infection and non-infection group.

**Conclusion:**

Our data revealed that increased available and high-quality embryo rates seen in the non-infection group during second COS cycle were not observed in infected patients. Nonetheless, when comparing intra-individual, no detrimental effects on laboratory or clinical outcomes were found. This indicates that while COVID-19 appears to compromise the advantages of a repeat cycle, it does not worsen ovarian function.

## 1 Introduction

Coronavirus Disease 2019 (COVID-19), caused by the severe acute respiratory syndrome coronavirus 2 (SARS-CoV-2), has evolved into a global pandemic. Despite the outbreak occurred more than 3 years ago, the disease continues to persist. In China, a huge number of individuals have experienced their second or even third COVID-19 infection, despite the majority having received at least one dose of vaccine. Furthermore, during December 2022 and January 2023, Shanghai encountered a widespread outbreak that affected nearly its entire population, further heightening public concern regarding the virus. The abrupt relaxation of COVID-19 lockdown in Shanghai resulted in immediate and simultaneous large-scale infections among its residents. This scenario offers a valuable model for investigating the impact of the same viral strain on infection outcomes.

Concerns regarding the potential impact of COVID-19 on female reproductive health have led to hesitancy in pursuing fertility treatments following infection. Emerging evidence indicates that SARS-CoV-2 can interfere with key reproductive processes, including folliculogenesis, oocyte maturation, and ovulation, through interaction with cellular receptors like angiotensin-converting enzyme 2 (ACE2) (1). Notably, ACE2 are expressed in both granulosa cells and follicular fluid throughout every phase of follicular development in the human ovary (1, 2). This widespread expression indicates that SARS-CoV-2 could potentially disrupt female fertility at various stages.

To date, the impact of COVID-19 on female fertility remains controversial. Although some research report no significant adverse effects on oocyte quality, blastocyst development, or clinical pregnancy outcomes (3, 4), others have observed a decline in embryo quality, implantation rate and pregnancy rate, and higher rate of early pregnancy loss and cesarean deliveries (5–8). These conflicting findings underscore the complex relationship between SARS-CoV-2 infection and female reproductive function. Notably, existing studies comparing infected and uninfected cohorts often exhibit significant baseline heterogeneity, thereby introducing potential confounding factors that may compromise the validity of the observed associations. Moreover, differences in both timing of infection and viral subspecies across studies could also contribute to the discrepant outcomes, given that distinct variants differ in virulence, transmissibility, and interactions with the host immune system (9–14). These differences could potentially lead to variant-specific effects on the reproductive system. Additionally, longitudinal studies comparing the same patients before and after infection remain scarce in the current literature.

In light of the aforementioned considerations, we employed a before-and-after self-control retrospective study aiming to minimize bias arising from variations in patient background. Given the limitations of previous research, this study aims to evaluate the impact of COVID-19 on assisted reproductive technology (ART) laboratory and clinical outcomes through rigorous intra-individual comparisons in a population with normal ovarian reserve. In this study, we specifically enrolled participants who were infected between December 1, 2022 and January 31, 2023, sharing the same Omicron variant and receiving similar treatments, thereby minimizing population bias. We hope to provide valuable insights to assist patients in making well-informed decisions.

## 2 Methods and materials

### 2.1 Study design and inclusion criteria

This retrospective cohort study analyzed data from patients who had experienced controlled ovarian stimulation (COS) and oocyte pick-up (OPU) at the International Peace Maternity and Child Health Hospital of China Welfare Institute (IPMCH) between January 1st, 2022 and December 31st, 2023. The inclusion criteria were: (i) women under 40 years old; (ii) women with basal serum FSH levels below 10 IU/L; (iii) women who underwent two COS cycles using antagonist protocol without other adjuvant treatments (e.g., growth hormone, antioxidants, immunotherapy). Exclusion criteria: (i) women with multiple COVID-19 infections; (ii) women comorbid with severe systemic diseases potentially affecting conception. Participants who infected with COVID-19 between two cycles were categorized as the infected group, while those who had no history of infection were designated as the uninfected group.

All the infected patients were diagnosed by antigen or PCR test, which was noted in their medical records. Every patient had experienced routine serum SARS-CoV-2 antibody (IgG and IgM) tests and PCR tests for detecting SARS-CoV-2 RNA before the COS procedure in our center. If the initial antigen test is positive, retesting is required to confirm a positive diagnosis. All detection methods were the same as described in the previous study.

The ART data were systematically extracted from our institution’s electronic medical records database. All data we used in this study was secondary data and did not contain any data that could identify individual. The study qualified for ethical approval exemption. All the personal information was kept confidential.

### 2.2 Controlled ovarian stimulation protocols

The gonadotropin-releasing hormone (GnRH) antagonist protocol was implemented according to established methods (15, 16). Briefly, daily subcutaneous injections of ganirelix acetate (0.25 mg, Chiatai Tianqing, Jiangsu, China) were initiated when at least one follicle reached 13–14 mm in diameter measured by transvaginal ultrasound to suppress the pituitary function. The recombinant follicle-stimulating hormone (rFSH) (Luveris®, Merck Serono, Geneva, Switzerland) dosage and stimulation duration were adjusted based on hormone level and ovarian response. Recombinant human chorionic gonadotropin (r-hCG, 250 μg Ovidrel®, Merck Serono, Geneva, Switzerland) was injected subcutaneous to trigger final oocyte maturation, when two to three dominant follicles reached a mean diameter of ≥18 mm. Oocytes were retrieved 36 h post-trigger guided by transvaginal ultrasound. Cumulus-oocyte complex maturity was assessed morphologically through evaluation of granulosa cell expansion patterns. Post-retrieval, fertilization via *in vitro* fertilization (IVF) or intracytoplasmic sperm injection (ICSI) was conducted as per semen analysis outcomes. Embryo quality was systematically evaluated from day 2 to 6 post-retrieval, utilizing the Cummins classification system for cleavage-stage embryos (days 2–3) and the Gardner-Schoolcraft grading criteria for blastocyst-stage embryos (days 5–6).

### 2.3 Definitions of study outcomes

The primary outcome mainly focused on the proportion of high-quality embryos per cycle. The secondary outcomes included oocytes retrieval, the rate of mature (MII) oocytes, fertilization rate, embryo cleavage rate, and the embryo yield per cycle. High-quality embryos were characterized as grade I or II with 6 to 10 cells on the third day, or as grade 3BB or higher on the fifth or sixth day.

### 2.4 Statistical analysis

In this study, we conducted statistical analyses utilizing SPSS software, version 26.0, from IBM Inc. USA, without filling in any missing data. We reported continuous data as the mean along with the standard deviation, or as the median with the interquartile range, while categorical data were displayed in terms of frequency counts. For comparing groups, we employed the *t*-test or one-way ANOVA for continuous variables as needed. Independent Student’s *t*-test was used to compare the infected group with the infected group, while paired *t*-test was used to compare the parameters before and after infection. In the case of categorical variables, we used the Pearson chi-square test, opting for Fisher’s exact test when expected counts were below 5 or when the total sample size was under 40. A *p*-value under 0.05 was accepted as indicative of statistical significance.

## 3 Results

This strictly self-controlled study enrolled 134 patients who met the inclusion criteria between January 1, 2022 and December 31, 2023. Among them, 68 patients remained uninfected during both their first and second COS cycles, while 66 patients contracted COVID-19 between their first and second COS cycles (Table 1). All enrolled patients were either asymptomatic or mild symptoms. No significant differences were observed between the infected and non-infected group in terms of mean age, duration of infertility, reproductive history (prior pregnancy and childbirth), baseline hormone levels, anti-Müllerian hormone (AMH) levels, or causes of infertility. The median interval from COVID-19 infection to the subsequent OPU was 79.5 days.

**Table 1:** Baseline characteristics of patients.

Characteristics	Non-infection	Infection	*p*-value
Number of patients	68	66	–
Age, years (mean ± SD)	32.85 ± 3.61	32.23 ± 3.23	0.254
BMI, kg/m^2^ (mean ± SD)	22.46 ± 3.13	22.25 ± 3.29	0.812
Infertility Duration, years (mean ± SD)	2.38 ± 1.93	2.81 ± 2.25	0.210
Gravidity, *n* (%)			0.952
0	42 (61.76%)	40 (60.61%)	–
1	15 (22.06%)	16 (24.24%)	–
≥2	11 (16.18%)	10 (15.15%)	–
Parity, *n* (%)			
0	59 (86.76%)	61 (92.42%)	0.284
≥1	9 (13.24%)	5 (7.58%)	–
Basal FSH, IU/L (mean ± SD)	7.34 ± 1.42	6.91 ± 1.49	0.109
Basal LH, IU/L (mean ± SD)	4.65 ± 3.05	4.69 ± 3.39	0.973
Basal estradiol, pmol/L (mean ± SD)	136.93 ± 44.59	141.93 ± 60.15	0.570
Basal progesterone, nmol/L (mean ± SD)	2.03 ± 2.60	1.83 ± 1.79	0.609
Basal AMH, ng/mL (mean ± SD)	3.56 ± 2.11	4.24 ± 1.90	0.055
Cause of Infertility, *n* (%)			0.656
Tubal	41 (60.29%)	47 (71.21%)	–
Male	13 (19.12%)	10 (15.15%)	–
Ovulatory disorder	4 (5.88%)	4 (6.06%)	–
Uterine factor	2 (2.94%)	1 (1.52%)	–
Unexplained	8 (11.76%)	4 (6.06%)	–
Interval between COVID-19 infection and post-infection OPU, days [median (Q1, Q3)]	–	79.50 (31.75, 150.25)	–

AMH, Anti-Mullerian hormone; BMI, Body mass index; COVID-19, Coronavirus Disease 2019; FSH, follicle-stimulating hormone; LH, luteinizing hormone; Q1, first quartile; Q3, third quartile.

**Figure 1:**
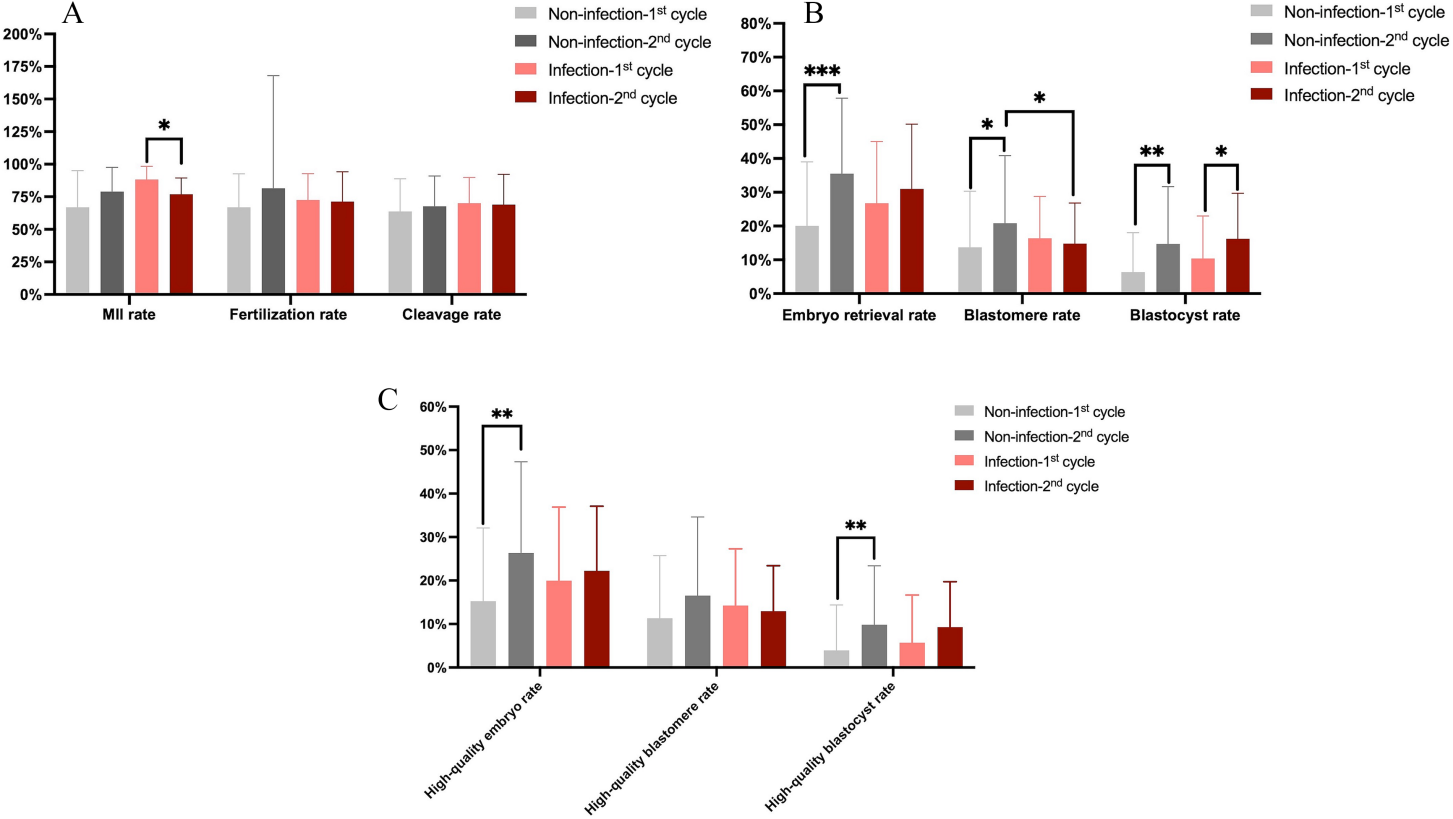
Comparison of COS-related laboratory parameters. **(A)** Comparison of fertilization rate and cleavage rate. **(B)** Comparison of total embryo rate, available cleavage stage, and blastocyst stage embryo rate. **(C)** Comparison of high-quality embryo rate, high-quality cleavage stage, and blastocyst stage embryo rate. * Represents *p* < 0.05; ** represents *p* < 0.01; *** represents *p* < 0.001.

The laboratory parameters of COS cycles are summarized in Table 2 and Figure 1. Both the infection and non-infection groups showed comparable results between their first and second COS cycles in terms of the duration of gonadotropin (Gn) stimulation, total Gn dosage, and endometrial thickness on the day of hCG administration. In the non-infection group, the E2 level on the hCG trigger day was significantly higher in the second cycle than in the first (*p* = 0.037). No such difference was observed in the infection group. The proportion of cycles utilizing ICSI for fertilization increased in the second cycle for both groups. This adjustment made in the second cycle was likely as a compensation measure in case of the poor sperm motility and low fertilization rate during the first cycle using IVF (Supplementary Table S1). Notably, while the MII oocyte rate remained comparable between the two cycles in the non-infection group, it significantly decreased in the second (post-infection) cycle of the infection group (*p* = 0.006).

**Table 2:** Comparison of COS-related laboratory parameters pre- or post-COVID-19 infection.

Characteristics	Non-infection Group		*p*-value	Infection group		*p*-value	*p* value (2nd COS cycle of two groups)
	1st COS cycle	2nd COS cycle		1st COS cycle	2nd COS cycle		
Duration of stimulation, days (mean ± SD)	9.07 ± 1.25	9.66 ± 1.62	**0.019**	9.88 ± 1.67	9.80 ± 1.90	0.808	0.643
Range, days	6–12	7–14	–	5–15	3–16	–	–
Total gonadotropins dosage, U (mean ± SD)	20048.20 ± 589.64	2553.86 ± 2527.16	0.110	2076.52 ± 197.17	2157.27 ± 696.47	0.476	0.221
Estradiol levels, pmol/L (mean ± SD)	99737.77 ± 5539.28	12275.26 ± 8262.12	**0.037**	13084.23 ± 8530.69	14072.28 ± 9342.38	0.527	0.240
Endometrial thickness, mm (mean ± SD)	10.33 ± 2.18	10.21 ± 2.16	0.756	9.99 ± 2.52	10.15 ± 2.51	0.717	0.884
Fertilization methods, n (%)			**0.001**			**0.004**	
IVF	47 (69.12%)	27 (39.71%)	–	54 (81.82%)	39 (59.09%)	–	–
ICSI	21 (30.88%)	41 (60.29%)	–	12 (18.18%)	27 (40.91%)	–	–
Total number of oocytes retrieved	685	799		819	941	–	–
Oocyte yield per cycle, n (mean ± SD)	10.07 ± 5.23	11.75 ± 7.32	0.128	12.41 ± 6.90	14.26 ± 8.49	0.172	0.069
Range	0–6	1–34	–	3–35	1–38	–	–
Number of MII oocytes (mean ± SD)	6.23 ± 3.66	8.23 ± 5.23	0.134	10.43 ± 5.79	12.26 ± 7.01	0.411	**0.009**
MII/oocytes, % (mean±SD) (ICSI only)	66.94 ± 28.20	78.93 ± 18.65	0.077	88.24 ± 10.16	76.92 ± 12.52	**0.006**	0.626
Fertilization rate, % (mean ± SD)	66.99 ± 25.61	81.47 ± 86.48	0.188	72.62 ± 20.18	71.24 ± 22.88	0.714	0.354
Cleavage rate, % (mean ± SD)	63.75 ± 25.02	67.66 ± 23.28	0.348	70.09 ± 19.77	68.98 ± 23.25	0.769	0.743
Total embryo yield, n	118	234	–	178	255	–	–
Blastomere	77 (65.25%)	108 (46.15%)	–	102 (57.30%)	105 (41.18%)	–	–
Blastocyst	41 (34.75%)	126 (58.85%)	–	77 (42.70%)	150 (58.82%)	–	–
Embryo yield per cycle, n (mean ± SD)	1.74 ± 1.36	3.44 ± 2.59	**0.000**	2.71 ± 1.61	3.86 ± 2.39	**0.001**	0.329
Blastomere	1.13 ± 1.11	1.59 ± 0.95	**0.011**	1.55 ± 0.83	1.59 ± 0.80	0.749	0.986
Blastocyst	0.60 ± 1.02	1.85 ± 2.62	**0.000**	1.17 ± 1.47	2.27 ± 2.18	**0.001**	0.316
Embryo retrieval rate, % (mean ± SD)	20.06 ± 18.96	35.50 ± 22.37	**0.000**	26.76 ± 18.27	31.02 ± 19.17	0.194	0.215
Blastomere	13.69 ± 16.64	20.81 ± 20.04	**0.026**	16.38 ± 12.42	14.78 ± 12.05	0.453	**0.037**
Blastocyst	6.36 ± 11.64	14.69 ± 16.98	**0.001**	10.39 ± 12.57	16.24 ± 13.49	**0.011**	0.561
Total high-quality embryo yield, n	87	174	–	126	187	–	–
Blastomere	62 (71.26%)	89 (51.15%)	–	87 (69.05%)	97 (51.87%)	–	–
Blastocyst	27 (28.74%)	85 (48.85%)	–	39 (30.95%)	90 (48.13%)	–	–
High-quality embryo yield per cycle, n (mean ± SD)	1.28 ± 1.22	2.56 ± 2.62	**0.000**	1.91 ± 1.34	2.83 ± 1.97	**0.002**	0.456
Blastomere	0.91 ± 0.097	1.31 ± 1.01	**0.021**	1.32 ± 0.83	1.47 ± 0.83	0.294	0.316
Blastocyst	0.37 ± 0.88	1.25 ± 2.03	**0.001**	0.59 ± 1.12	1.36 ± 1.70	**0.003**	0.726
High-quality embryo rate, % (mean ± SD)	15.24 ± 16.83	26.34 ± 20.97	**0.001**	19.98 ± 16.90	22.24 ± 14.86	0.416	0.194
Blastomere	11.31 ± 14.41	16.54 ± 18.07	0.064	14.26 ± 13.03	12.96 ± 10.44	0.527	0.164
Blastocyst	3.94 ± 10.46	9.81 ± 13.58	**0.006**	5.71 ± 10.96	9.28 ± 10.46	0.058	0.801

ICSI, intracytoplasmic sperm injection; IVF, in-vitro fertilization; SD, standard deviation. Bold font indicates *P* < 0.05.

In the non-infection group, the fertilization rate, cleavage rate, available embryo rate, and high-quality embryo rate were all higher in the second cycle than in the first, though the increases in fertilization and cleavage rates did not reach statistical significance. When available embryos were analyzed separately by developmental stage, both cleavage-stage and blastocyst-stage embryo rates improved in the second cycle, with the increase in blastocyst formation rate being more pronounced (*p* = 0.001). We also separated the high-quality blastomeres and blastocysts, no significant difference was observed in the rate of high-quality cleavage embryos between cycles; however, the high-quality blastocyst rate was significantly increased in the second cycle (*p* = 0.006). These findings suggest that physicians may optimize the stimulation protocol in a subsequent COS cycle based on insights gained from the first cycle, thereby effectively improving embryo yield and quality—particularly in enhancing blastocyst development potential.

In the infection group, no statistically significant differences were observed in the fertilization rate or cleavage rate between the first and second COS cycles. In the infection group, the oocyte retrieval rate of the second cycle was higher than that of the first cycle, but the difference was not statistically significant (*p* = 0.194). Similarly, the blastomere formation rate showed no notable change. However, the blastocyst formation rate was significantly elevated in the post-infection (second) cycle compared to the pre-infection cycle (*p* = 0.011). Despite this, the high-quality embryo rate did not differ significantly between the two cycles. Collectively, these results indicate that undergoing a second COS cycle after COVID-19 infection did not lead to an overall improvement in embryo developmental competence, which may be attributed to the adverse effects of the viral infection.

We further compared the corresponding parameters of each cycle between the infection and non-infection groups. The results indicated that all assessed parameters were comparable between the two groups during the first cycle. In the second cycle, however, the blastomere rate was significantly lower in the infection group than in the non-infection group (*p* = 0.037).

**Figure 2:**
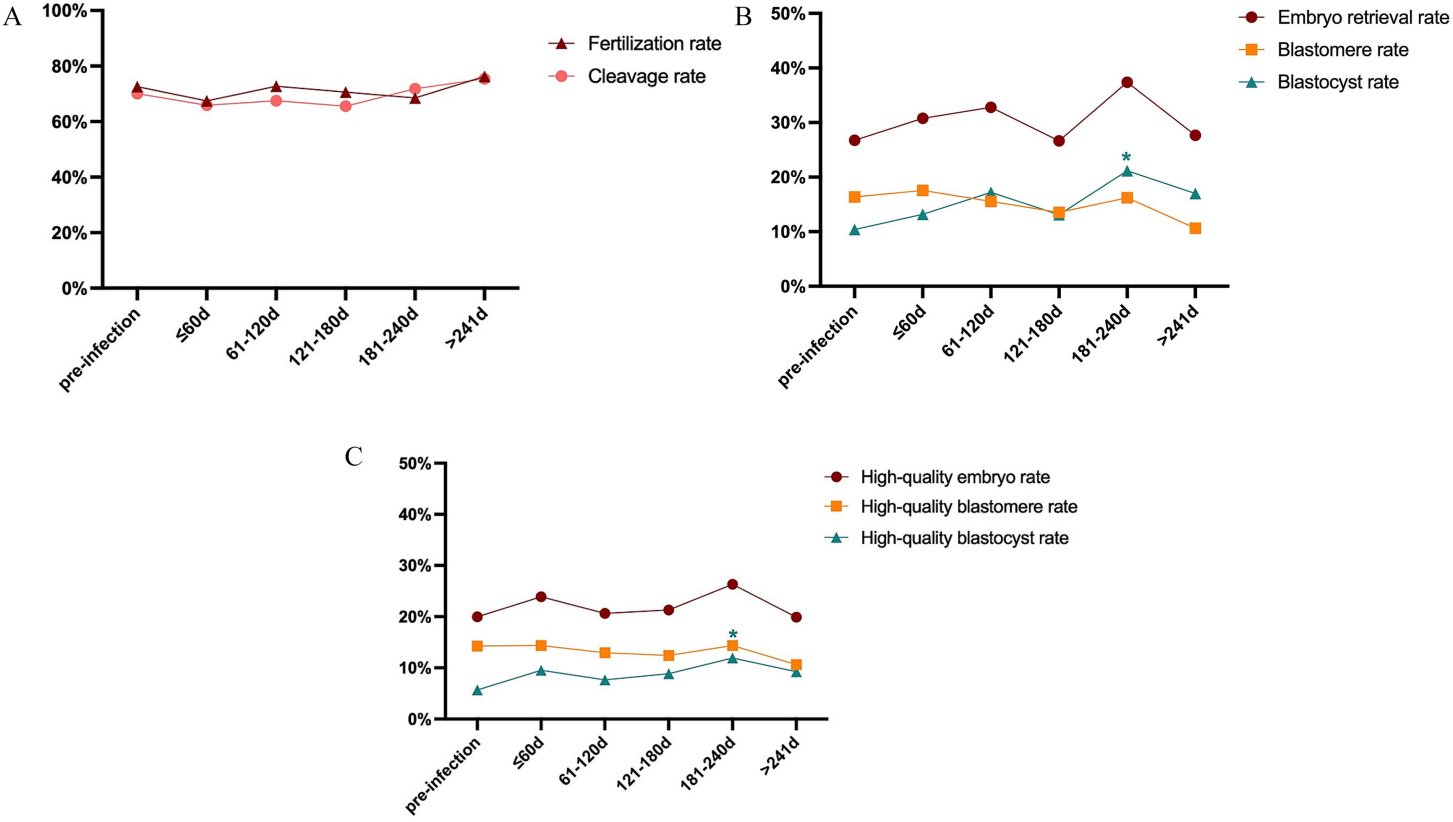
Subgroup analysis of laboratory outcomes pre/post-infection. **(A)** Comparison of fertilization rate and cleavage rate. **(B)** Comparison of total embryo rate, available cleavage stage, and blastocyst stage embryo rate. **(C)** Comparison of high-quality embryo rate, high-quality cleavage stage, and blastocyst stage embryo rate.

The time interval between COVID-19 infection and the start of ART treatment is important for clinical decision-making. As a highlight of this article, we stratified the infection group into 5 subgroups according to the intervals between infection and post-infection OPU day: ≤ 60 days, 61–120 days, 121–180 days, 181–240 days, >241 days (Table 3; Figure 2). The oocyte yields, fertilization rate, cleavage rate, total embryo retrieval rate, blastocyst formation rate, and high-quality embryo rate pre−/ post-infection were compared. We observed increased rate of total and high-quality blastocyst in the subgroup of 181–240 days after infection. None of the other subgroups showed significance of the above parameters. Moreover, to better investigated the time related influence of COVID-19 infection, we also stratified the infection group by shorter intervals, namely 9 groups: ≤ 30 days, 31–60 days, 61–90 days, 91–120 days, 121–150 days, 151–180 days, 181–240 days, 241–360 days, and >360 days (Supplementary Table S2; Supplementary Figure S1). The results showed no significance difference between subgroups except for the subgroup of 181–240 days, which was similar with the above analysis. Overall, taking the time interval between COVID-19 infection and ART treatment initiation into account, the prior COVID-19 infection had no significant adverse effects on oocyte quality or embryonic development potential.

**Table 3:** Subgroup analysis of laboratory outcomes pre/post-infection.

Characteristics	Pre-infection	Intervals between COVID-19 infection and post-infection OPU				
	*n* = 66	≤60d *n* = 13	61-120d *n* = 17	121-180d *n* = 13	181-240d *n* = 11	>241d *n* = 12
Fertilization rate, %						
mean ± SD	72.62 ± 20.18	67.47 ± 26.70	72.77 ± 18.34	70.65 ± 24.04	68.53 ± 29.31	76.27 ± 18.92
*p*-value	–	0.438	0.979	0.766	0.566	0.595
Cleavage rate, %						
mean ± SD	70.09 ± 19.77	65.92 ± 27.08	67.54 ± 21.83	65.57 ± 27.57	71.81 ± 22.35	75.39 ± 18.35
*p*-value	–	0.53	0.669	0.496	0.808	0.438
Embryo retrieval rate, %						
mean ± SD	26.76 ± 18.27	30.77 ± 24.52	32.77 ± 16.51	26.65 ± 19.90	37.39 ± 18.95	27.65 ± 16.72
*p*-value	–	0.484	0.242	0.984	0.086	0.880
Blastmere rate, %						
mean ± SD	16.38 ± 12.42	17.58 ± 16.79	15.55 ± 10.62	13.55 ± 9.798	16.20 ± 14.06	10.64 ± 8.299
*p*-value	–	0.748	0.806	0.451	0.967	0.141
Blastocyst rate, %						
mean ± SD	10.39 ± 12.57	13.19 ± 13.53	17.22 ± 9.513	13.10 ± 12.88	21.18 ± 20.85	17.00 ± 10.83
*p*-value	–	0.481	0.057	0.495	**0.012**	0.109
High-quality embryo rate, %						
mean ± SD	19.98 ± 16.90	23.91 ± 15.41	20.66 ± 13.12	21.32 ± 17.70	26.32 ± 18.31	19.90 ± 10.82
*p*-value	–	0.422	0.876	0.783	0.228	0.989
High-quality blastomere rate, %						
mean ± SD	14.26 ± 13.03	14.37 ± 9.893	12.97 ± 9.761	12.45 ± 10.48	14.39 ± 14.80	10.64 ± 8.299
*p*-value	–	0.976	0.692	0.618	0.974	0.337
High-quality blastocyst rate, %						
mean ± SD	5.7 ± 20.96	9.534 ± 9.929	7.689 ± 7.557	8.869 ± 11.65	11.93 ± 15.65	9.259 ± 8.601
*p*-value	–	0.247	0.504	0.339	**0.08**	0.299

OPU, oocyte pick-up; SD, standard deviation. Bold font indicates *P* < 0.05.

Finally, we compared the long-term clinical outcomes between the two groups. Since most patients underwent a second COS cycle due to either implantation failure or poor embryo quality in the first cycle, we only collected the pregnancy outcomes of the first embryo transfer (ET) following the second COS cycle. No significant differences were observed between the non-infection and infection groups in terms of biochemical pregnancy, clinical pregnancy, miscarriage, preterm birth, or full-term delivery rates (Table 4).

**Table 4:** Comparison of clinical outcomes between non-infection and infection groups.

Characteristics	Non-infection group	Infection group	*p*-value
Embryo transfer, *n*	49	59	0.753
Fresh ET, *n* (%)	8 (16.33%)	11 (18.64%)	–
Frozen ET, *n* (%)	41 (83.67%)	48 (81.36%)	–
Biochemical pregnancy, *n* (%)	38 (77.55%)	40 (67.80%)	0.260
Clinical pregnancy, *n* (%)	31 (63.26%)	34 (57.63%)	0.883
Pregnancy outcomes			0.611
Miscarriage	7 (14.29%)	8/59	–
Premature birth	1 (2.04%)	3/59	–
Full-term delivery	23 (46.94%)	2/59	–

ET, Embryo transfer.

## 4 Discussion

Our findings demonstrated that initiating COS treatment after COVID-19 infection was not associated with compromised female fertility. Comparisons were made between infected and non-infected patients, as well as intra-individual comparisons of pre- and post-infection COS cycles in the same patients. To our knowledge, this is the first study to examine the impact of repeated COS cycles on embryo development in the context of COVID-19 infection. Although a lower blastomere rate was observed in the infection group during the second COS cycle, no significant intra-individual decline was detected between pre- and post-infection cycles within the same patients. Furthermore, neither the overall embryonic developmental potential nor subsequent pregnancy outcomes were significantly influenced by the interval between SARS-CoV-2 infection and COS treatment.

We observed decreased MII rate in infected patients undergoing ICSI. The SARS-CoV-2 invades cells through the ACE-2 receptor, a key player in the development of follicles (1). Studies have shown that during the periovulatory period and post-administration of human chorionic gonadotropin, both mRNA and protein levels of ACE2 increase in granulosa cells and dominant follicles, indicating the potential influence of virus on ovarian function and women’s fertility (2). Furthermore, it has been proposed that SARS-CoV-2 infection elevates arachidonic acid and inflammatory lipid mediators, triggering a cascade of systemic inflammatory responses (17). Such aberrant systemic inflammation, along with elevated oxidative stress following infection, could adversely affect oocyte maturation, fertilization, and subsequent embryo development (18, 19).

The impact of SARS-CoV-2 infection on ovarian reserve remained controversial. Herrero et al. (20) discovered that COVID-19 infection led to a decrease in the number of retrieved oocytes and adversely affected the follicular microenvironment. Similarly, Ding et al. (21) revealed lower AMH levels and elevated FSH levels in women following COVID-19 infection. In contrast, other studies suggest that mild SARS-CoV-2 infection may not compromise ovarian reserve or function, as indicated by stable levels of AMH, FSH, LH, and E2 (7, 22–24). Our self-controlled study supported the latter findings, showing no significant adverse effects on ovarian reserve, ovarian responses, or sex hormone levels after recovery from COVID-19.

Orvieto et al. (25) reported no impact on ovarian reserve in the subsequent IVF cycle for COVID-19-infected individuals, yet observed a reduction in the percentage of high-quality embryos. Another study also identified a reduction in oocyte and embryo quality compared to non-infected control group (26). In our study, while the blastomere rate was lower in the infection group comparing to non-infection group, other embryological parameters remained unaltered. Given that repeated COS cycles generally lead to improvements in oocyte and embryo quality, our findings suggest that SARS-CoV-2 infection may attenuate such cycle-to-cycle improvements.

Nevertheless, in our intra-individual comparison of cycles before and after SARS-CoV-2 infection, we observed no significant differences in oocyte yield, fertilization rate, cleavage rate, usable embryo rate, or the proportion of high-quality embryos. These results suggest that SARS-CoV-2 infection did not notably compromise ovarian function or embryo quality. Corroborating these findings, the largest prospective multicenter study to date similarly reported no adverse effects of prior infection on oocyte retrieval or embryological outcomes (27). Additional research showed no changes in oocyte yield, MII rate, fertilization success, 2PN formation, or number of cryopreserved embryos following infection, reinforcing this conclusion (23, 28).

The time interval between SARS-CoV-2 infection and ART treatment is a critical factor in clinical decision-making. To investigate its potential influence, we stratified patients by the duration from infection to COS treatment, considering that primordial follicles may be recruited up to 1 year in advance (29). Overall, our analysis revealed comparable laboratory outcomes before and after infection across subgroups. Notably, we observed an increase in the blastocyst formation rate at 181–240 days post-infection. Similarly, Hu et al. (30) reported significant increase in MII oocyte rate, fertilization rate, and high-quality blastocyst rate among patients with an interval of ≤3 months, whereas intervals beyond 3 months showed no significant changes. Chen et al. (27) observed a reduction in the number of 2PN zygotes and top-quality embryos when ART was performed within 1 week after infection. Meanwhile, Youngster et al. (5) identified a decline in oocyte yield among patients who underwent ART more than 180 days after SARS-CoV-2 infection. It is important to note that previous studies included patients with heterogeneous baseline characteristics, diverse viral variants, and varying COS protocols. In contrast, our study analyzed outcomes before and after infection within the same patients under consistent COS protocols, thereby minimizing inter-individual bias and enhancing the internal validity of our findings.

In this study, we observed no adverse effect of SARS-CoV-2 infection on pregnancy outcomes. Consistent with our findings, several studies have also reported no detrimental impact of COVID-19 infection in neither fresh ET cycles (4, 31, 32) nor frozen ET cycles (3, 33). Furthermore, even when infection occurred during IVF treatment, no significant decline in ongoing pregnancy rate was observed among infected women who did not develop high fever (34). Together, these findings offer valuable evidence regarding the limited influence of COVID-19 on female reproductive success.

This research had a few limitations. Firstly, it was a retrospective analysis and got its inherent bias within. Besides, the infection status of male partners was unknown due to lack of medical records and unclear memories. However, studies have reported no significant difference in the semen characteristics before versus after COVID-19 (35, 36). Moreover, one study have incorporated male semen analysis, IVF treatment outcomes and rates of early pregnancy loss appear unaffected by prior SARS-CoV-2 infection, despite minor reductions in sperm concentration observed among recently recovered individuals (4). Collectively, these findings suggest that SARS-CoV-2 infection has only little influence on semen quality and subsequent embryonic and clinical outcomes.

Our study also had some strengths. The primary one of our study lies in its before-and-after self-controlled cohort design, which minimized confounding from variables such as patient age, BMI, baseline health status, causes of infertility, and COS protocols. In addition, we exclusively included women with normal ovarian reserve, in contrast to earlier studies that involved poor ovarian responders (30), thereby improving the generalizability of our findings. Another notable advantage stems from the unique epidemiological context in Shanghai. Following the rapid lifting of lockdown measures in December 2022 and January 2023, a nearly simultaneous, large-scale outbreak of COVID-19 occurred among the local population. This setting provided a distinctive natural experiment for assessing the effects of the same viral subvariant on ART outcomes. Moreover, given the clinical relevance of the timing between SARS-CoV-2 infection and ART initiation, we stratified the infected cohort into five subgroups based on the interval from infection to post-infection oocyte pickup (OPU). This stratification allowed us to trace the dynamic impact of COVID-19 on ART results over time, thereby improving the temporal accuracy of our effect assessments.

## 5 Conclusion

Our findings indicate that while enhanced embryo viability and a higher proportion of high-quality embryos were observed in the second COS cycle among uninfected patients, no comparable improvements were seen in the infected group. However, when comparing post-infection outcomes to pre-infection parameters within the same individuals, SARS-CoV-2 infection did not exhibit adverse effects on female fertility in terms of ovarian response, laboratory outcomes, or pregnancy results. These results suggest that although infection may counteract some of the benefits associated with repeated COS cycles, it does not lead to a deterioration in ovarian reserve or ovarian responsiveness. Moreover, after accounting for the time interval between COVID-19 infection and the initiation of ART treatment, infection showed no significant detrimental impact on oocyte yield or embryo quality.

## Data Availability

The raw data supporting the conclusions of this article will be made available by the authors, without undue reservation.
