# Supplementary figures and images for: Impact of COVID-19 infection on laboratory and clinical outcomes of ovarian stimulation using antagonist protocol
Source: Front Med (Lausanne). 2025 Dec 19;12:1674189. doi: 10.3389/fmed.2025.1674189 (PMC12757384; doi:10.3389/fmed.2025.1674189)

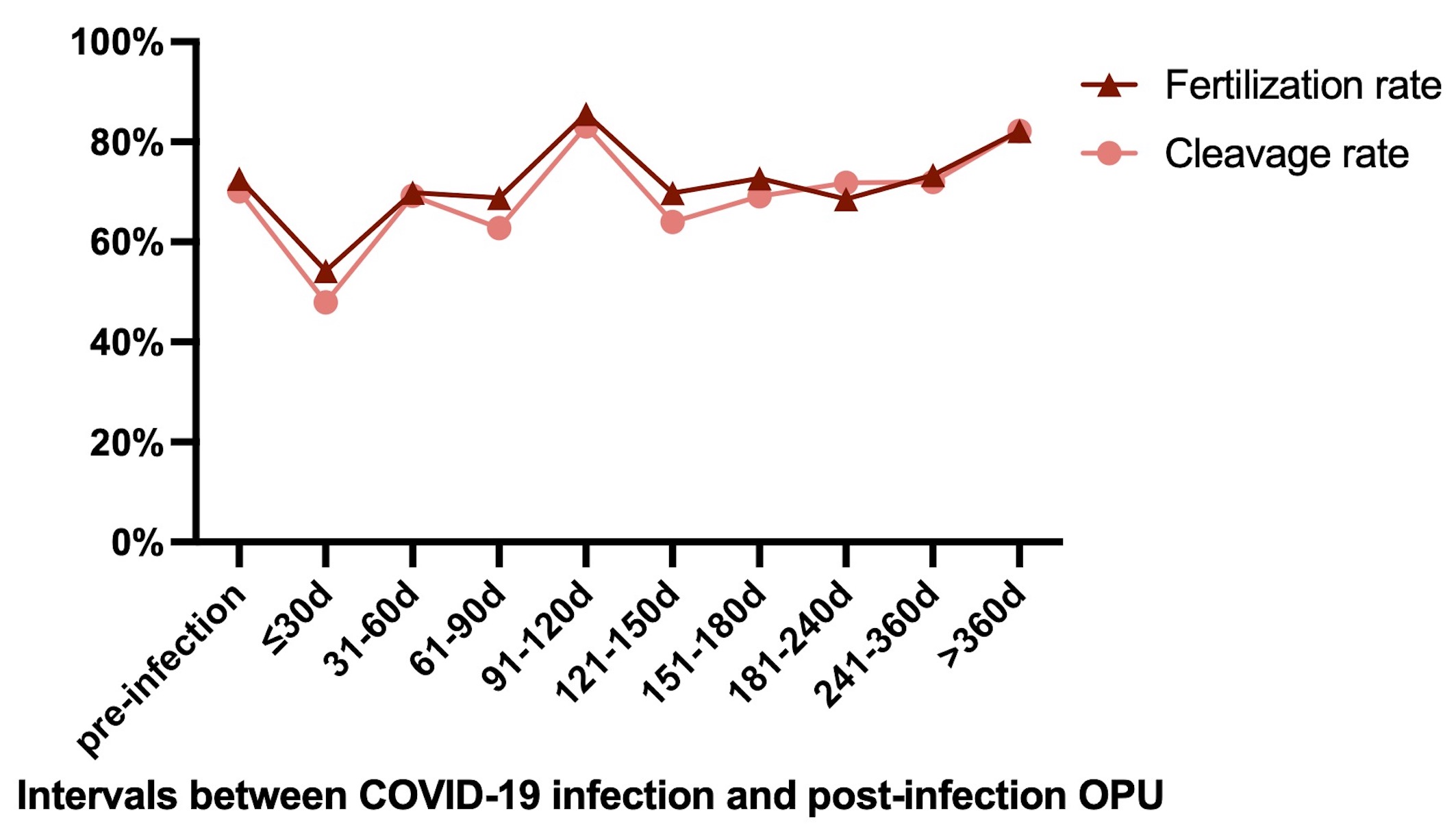

Supplement: Supplementary file 1 [file Image_1.JPEG]

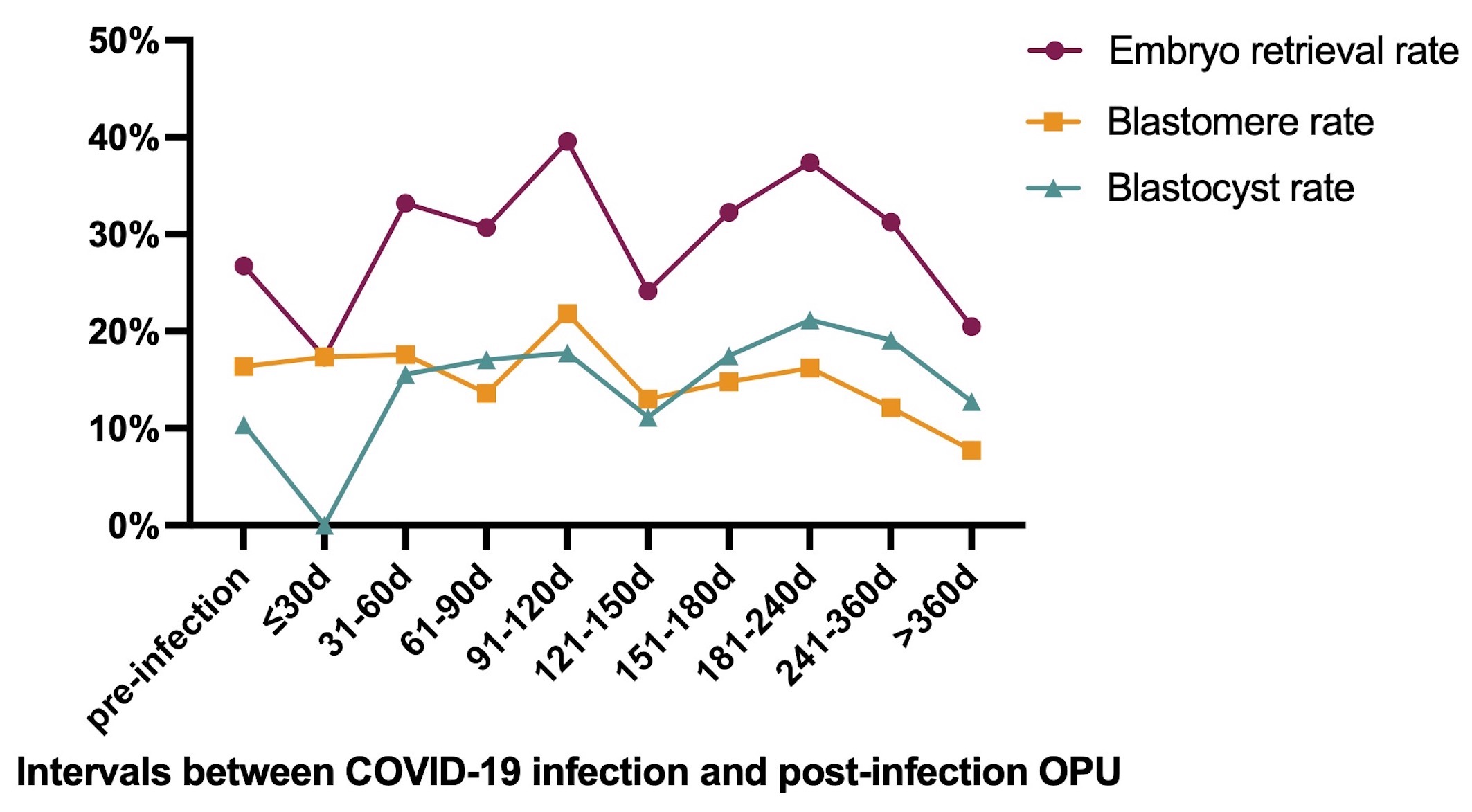

Supplement: Supplementary file 2 [file Image_2.JPEG]

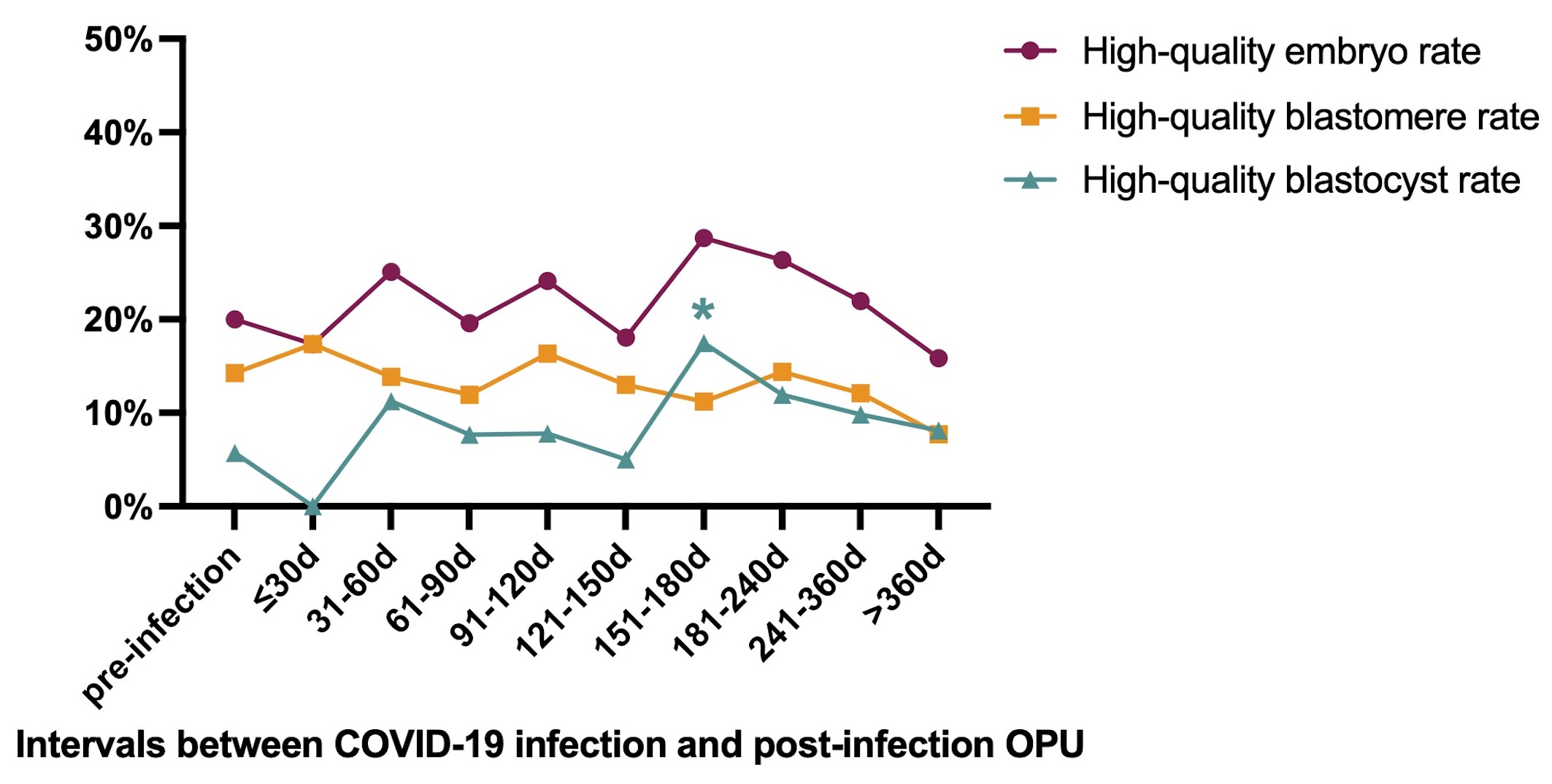

Supplement: Supplementary file 3 [file Image_3.JPEG]
