# Supplementary material for: Impact of COVID-19 infection on laboratory and clinical outcomes of ovarian stimulation using antagonist protocol
Source: Front Med (Lausanne). 2025 Dec 19;12:1674189. doi: 10.3389/fmed.2025.1674189 (PMC12757384; doi:10.3389/fmed.2025.1674189)
Supplement: Supplementary file 4 [file Table_1.DOCX]

**Impact of COVID-19 Infection on Laboratory and Clinical Outcomes of Ovarian Stimulation Using Antagonist Protocol**

Yu Yan^1,2#^, Mengxi Guo^1,2#^, Xiaojun Chen^1,2^, Qing Zhang^1,2^, Li Wang^1,2^, Meiling Zhang^1,2^, Min Wang^1,2^, Wen Li^1,2*^, Yu Tao^1,2*^

^1^ Center for Reproductive Medicine & Fertility Preservation Program, International Peace Maternity and Child Health Hospital, School of Medicine, Shanghai Jiao Tong University, Shanghai, 200030, China

^2^ Shanghai Key Laboratory of Embryo Original Diseases, Shanghai 200030, China

^#^ These authors contributed equally to the work and should be regarded as co-first authors.

* These authors contributed equally to this work and should be considered co-corresponding authors.

*** Correspondence:**Corresponding Author
Author Name: Wen Li

Academic Affiliation: Center for Reproductive Medicine & Fertility Preservation Program, International Peace Maternity and Child Health Hospital, School of Medicine, Shanghai Jiao Tong University

Mailing Address: Center for Reproductive Medicine & Fertility Preservation Program, International Peace Maternity and Child Health Hospital, School of Medicine, Shanghai Jiao Tong University, Shanghai, 200030, China

Phone: +86 13601858540

Email: [liwen@shsmu.edu.cn](mailto:liwen@shsmu.edu.cn)

Author Name: Yu Tao

Academic Affiliation: Center for Reproductive Medicine & Fertility Preservation Program, International Peace Maternity and Child Health Hospital, School of Medicine, Shanghai Jiao Tong University

Mailing Address: Center for Reproductive Medicine & Fertility Preservation Program, International Peace Maternity and Child Health Hospital, School of Medicine, Shanghai Jiao Tong University, Shanghai, 200030, China

Phone: +86 15901656819

Email: [taoy2010@163.com](mailto:taoy2010@163.com)

**Keywords:** COVID-19, IVF outcome, embryonic development, ovarian response, Controlled ovarian stimulation, fertility evaluation

Supplementary Tables and Figures

**Supplementary Table S1** The IVF fertilization rate of the infected group/uninfected group in the first cycle

**Supplementary Table S2** Subgroup analysis of laboratory outcomes pre/post-infection by shorter intervals

**Supplementary Table S3** Comparison of semen parameters of the male partners of the infection group

**Supplementary Figure S1** Subgroup analysis of laboratory outcomes pre/post-infection by shorter intervals. (A) Comparison of fertilization rate and cleavage rate. (B) Comparison of total embryo rate, available cleavage stage, and blastocyst stage embryo rate. (C) Comparison of high-quality embryo rate, high-quality cleavage stage, and blastocyst stage embryo rate.

**Supplementary Table S1** The IVF fertilization rate of the infected group/uninfected group in the first cycle

|  | **Non-infection group** | **Infection group** | ***P*-value** |
| --- | --- | --- | --- |
| **Total** | 68.94±26.13 | 71.84±21.04 | 0.097 |
| **Patients switch to ICSI in second cycle** | 57.44±28.90 | 57.93±20.36 | 0.539 |
| ***P*-value** | 0.115 | 0.026 | - |

**Supplementary Table S2** Subgroup analysis of laboratory outcomes pre/post-infection by shorter intervals

| **Characteristics** | **Pre-infection** | **Intervals between COVID-19 infection and post-infection OPU** | | | | | | | | |
| --- | --- | --- | --- | --- | --- | --- | --- | --- | --- | --- |
|  | **n=66** | **≤30d**  **n=2** | **31-60d**  **n=11** | **61-90d**  **n=13** | **91-120d**  **n=4** | **121-150d**  **n=9** | **151-180d**  **n=4** | **181-240d**  **n=11** | **241-360d**  **n=8** | **>360d**  **n=4** |
| Fertilization rate, % | | | | | | | | | | |
| mean±SD | 72.62±20.18 | 54.17±29.46 | 69.89±26.96 | 68.83±18.76 | 85.60±10.01 | 69.74±25.42 | 72.69±24.12 | 68.54±28.32 | 73.34±20.98 | 74.23±63.96 |
| *P*-value | - | 0.242 | 0.702 | 0.569 | 0.252 | 0.712 | 0.995 | 0.567 | 0.931 | 0.399 |
| Cleavage rate, % | | | | | | | | | | |
| mean±SD | 70.09±19.77 | 47.92±20.62 | 69.20±27.59 | 62.76±22.12 | 83.10±12.75 | 64.00±28.06 | 69.12±30.28 | 71.81±22.36 | 72.02±19.94 | 65.71±24.16 |
| *P*-value | - | 0.157 | 0.901 | 0.268 | 0.246 | 0.431 | 0.931 | 0.807 | 0.812 | 0.282 |
| Embryo retrieval rate, % | | | | | | | | | | |
| mean±SD | 26.76±18.27 | 17.36±6.87 | 33.21±25.97 | 30.69±16.11 | 39.58±18.30 | 24.15±20.36 | 32.28±20.47 | 37.39±18.96 | 31.24±17.64 | 27.78±22.07 |
| *P*-value | - | 0.489 | 0.297 | 0.495 | 0.190 | 0.698 | 0.572 | 0.087 | 0.528 | 0.521 |
| Blastmere rate, % | | | | | | | | | | |
| mean±SD | 16.38±12.42 | 17.36±6.87 | 17.62±18.27 | 13.63±11.03 | 21.82±6.84 | 13.00±11.06 | 14.80±7.40 | 16.21±14.07 | 12.11±8.98 | 17.25±18.69 |
| *P*-value | - | 0.912 | 0.759 | 0.467 | 0.397 | 0.445 | 0.805 | 0.967 | 0.360 | 0.179 |
| Blastocyst rate, % | | | | | | | | | | |
| mean±SD | 10.39±12.57 | 0.0 | 15.59±13.37 | 17.05±7.29 | 17.76±16.42 | 11.16±11.28 | 17.48±16.97 | 21.18±20.86 | 19.13±10.95 | 10.53±15.10 |
| *P*-value | - | 0.271 | 0.225 | 0.096 | 0.277 | 0.869 | 0.295 | **0.013** | 0.077 | 0.724 |
| High-quality embryo rate, % | | | | | | | | | | |
| mean±SD | 19.98±16.90 | 17.36±6.87 | 25.10±16.43 | 19.60±12.17 | 24.10±17.51 | 18.04±13.79 | 28.70±25.32 | 26.33±18.32 | 21.93±10.57 | 20.80±19.75 |
| *P*-value | - | 0.822 | 0.333 | 0.940 | 0.622 | 0.737 | 0.298 | 0.231 | 0.747 | 0.622 |
| High-quality blastomere rate, % | | | | | | | | | | |
| mean±SD | 14.26±13.03 | 17.36±6.87 | 13.83±10.52 | 11.94±9.60 | 16.32±10.96 | 13.0±011.06 | 11.22±10.52 | 14.39±14.80 | 12.11±8.98 | 13.92±16.49 |
| *P*-value | - | 0.722 | 0.913 | 0.528 | 0.742 | 0.769 | 0.627 | 0.974 | 0.635 | 0.296 |
| High-quality blastocyst rate, % | | | | | | | | | | |
| mean±SD | 5.7±20.96 | 0.0 | 11.27±9.84 | 7.66±7.26 | 7.78±9.69 | 5.04±6.51 | 17.48±16.97 | 11.94±15.65 | 9.83±8.57 | 6.87±12.43 |
| *P*-value | - | 0.461 | 0.116 | 0.552 | 0.710 | 0.862 | **0.036** | 0.079 | 0.309 | 0.664 |

OPU=oocyte pick-up; SD=standard deviation

**Supplementary Table S3** Comparison of semen parameters of the male partners of the infection group

|  | First COS cycle | Second COS cycle | *P*-value |
| --- | --- | --- | --- |
| Volume (ml), mean± SD | 2.58±1.09 | 3.16±3.07 | 0.202 |
| Sperm concentration (mil/ml), median (min-max) | 27.52  (0.39 - 163.55) | 57.94  (2.78 - 257.5) | 0.590 |
| Total sperm number (million), median (min-max) | 72.37  (1.97 - 408.88) | 136.23  (6.39 - 644.83) | 0.006 |
| Progressive motility (%), mean± SD | 44.09±15.80 | 43.12±17.97 | 0.783 |
| Total motility (%), mean± SD | 52.89±16.59 | 50.09±19.91 | 0.464 |
| Immotility (%), mean± SD | 47.11±16.59 | 48.64±20.61 | 0.693 |
| Normal morphology (%), mean± SD | 3.08±1.40 | 3.58±3.52 | 0.348 |


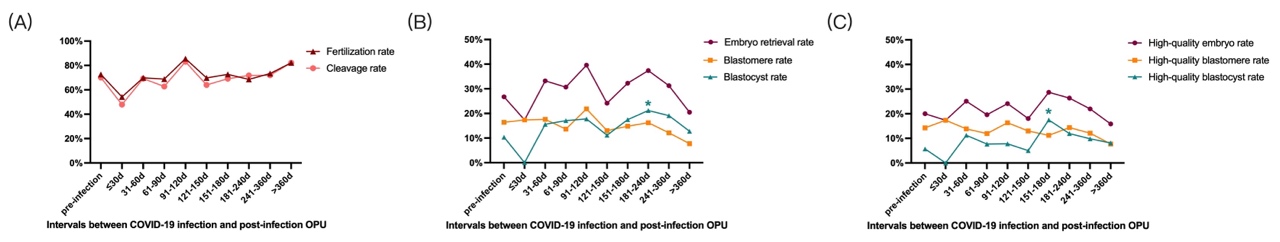


**Supplementary Figure S1** Subgroup analysis of laboratory outcomes pre/post-infection by shorter intervals. (A) Comparison of fertilization rate and cleavage rate. (B) Comparison of total embryo rate, available cleavage stage, and blastocyst stage embryo rate. (C) Comparison of high-quality embryo rate, high-quality cleavage stage, and blastocyst stage embryo rate.
